# Supplementary material for: Formation of high-aspect-ratio nanocavity in LiF crystal using a femtosecond X-ray free-electron laser pulse
Source: Nat Commun. 2025 Dec 17;16:11504. doi: 10.1038/s41467-025-66481-6 (PMC12749636; doi:10.1038/s41467-025-66481-6)
Supplement: Supplementary file 1 — Supplementary Information [file 41467_2025_66481_MOESM1_ESM.pdf]

Supplementary Information for

Formation of high-aspect-ratio nanocavity in LiF crystal using a femtosecond X-ray free-electron laser pulse

Sergey S. Makarov,*[1] Vasily V. Zhakhovsky,[1] Sergey Yu. Grigoryev,[1] Petr Chuprov,[2] Tatiana A. Pikuz,[3] Nail A. Inogamov,[1,4] Victor A. Khokhlov,[1,4], Yuri V. Petrov,[4] Evgeniy A. Perov,[1] Vadim Shepelev,[2] Takehisa Shobu,[5] Aki Tominaga,[5] Ludovic Rapp,[6] Saulius Juodkazis,[7,8] Mikako Makita,[9] Motoaki Nakatsutsumi,[9] Thomas R. Preston,[9] Karen Appel,[9] Zuzana Konopkova,[9] Valerio Cerantola,[9,10] Erik Brambrink,[9] Jan-Patrick Schwinkendorf,[9] István Mohacsi,[9] Vojtech Vozda,[11] Vera Hajkova,[11] Tomas Burian,[11] Jaromir Chalupsky,[11] Libor Juha,[11] Norimasa Ozaki,[12] Ryosuke Kodama,[12,13] Ulf Zastrau,[9] Andrei V. Rode[6] and Sergey A. Pikuz[14]

[1]Joint Institute for High Temperatures of Russian Academy of Sciences, 13/2 Izhorskaya st., 125412 Moscow, Russia
[2]Institute for Computer Aided Design, Russian Academy of Sciences, Moscow, 123056 Russia
[3]Institute for Open and Transdisciplinary Research Initiatives, Osaka University, Suita, 565-0871, Osaka, Japan
[4]Landau Institute for Theoretical Physics of Russian Academy of Sciences, 1-A Akademika Semenova av., Chernogolovka, Moscow Region, 142432, Russia
[5]The facility at Material Science Research Center, Japan Atomic Energy Agency, Sayo, Hyogo 679-5148, Japan
[6]Laser Physics Centre, Department of Quantum Science and Technology, Research School of Physics, Australian National University, Canberra ACT 2601, Australia
[7]Optical Sciences Centre and ARC Training Centre in Surface Engineering for Advanced Materials (SEAM), School of Science, Swinburne University of Technology, Hawthorn, VIC 3122, Australia
[8]Tokyo Tech World Research Hub Initiative (WRHI), School of Materials and Chemical Technology, Tokyo Institute of Technology, Tokyo 152-8550, Japan
[9]European XFEL, Holzkoppel 4, 22869 Hamburg, Germany
[10]Università degli Studi di Milano Bicocca, Piazza della Scienza 4, 20126 Milano, Italy
[11]Department of Radiation and Chemical Physics, Institute of Physics, Czech Academy of Sciences, Na Slovance 1999/2, 182 00 Prague 8, Czech Republic
[12]Graduate School of Engineering, Osaka University, Suita, 565-0871 Osaka, Japan
[13]Institute of Laser Engineering, Osaka University, Suita, 565-0871 Osaka, Japan
[14]HB11 Energy Holdings, Freshwater, NSW 2095, Australia

***Corresponding author:** Sergey Makarov
Email: seomakarov28@gmail.com

CONTENTS

I. Estimation of the threshold for crater and cavity formation in a LiF sample 2

2D hydrodynamic simulation approach 2

Simulation results..3

II. Mathematical model for LiF damage (SPH simulation).. 5

Material models..5

III. Interatomic potential for LiF in condensed phase (MD simulation)..................................... 9

IV. Supplementary References.. 11

I. Estimation of the threshold for crater and cavity formation in a LiF sample

2D hydrodynamic simulation approach

To estimate the thresholds for cratering and cavity opening, a computational algorithm based on Baer-Nunziato's system of equations[1], which uses a hydrodynamic approach to describe multiphase media, was used. Of course, the use of approximate equations for the state of aggregation and the lack of consideration of elastoplastic effects significantly limit the scope of the model for this type of problem, but the results obtained can be correlated qualitatively and quantitatively with experimental observations and computational results from other methods. The problem of the interaction of a rectangular beam with a LiF layer, without damping along the radius and deep into the substance, was considered.

To determine the threshold for crater formation and cavity opening, simulations were performed with irradiances in the range of absorbed energy densities of 10.5 kJ·cm^{-3} - 28 kJ·cm^{-3} with a step of 3.5 kJ·cm^{-3}. A uniform rectangular grid consisting of 500 cells in the r-direction and 300 cells in the z-direction was used for the simulations. The symmetry condition is used on the left edge of the computational domain and non-reflecting boundary conditions are used for the rest. The dimensions of the computational domain were chosen so that possible perturbations of the wave pattern by non-reflecting boundary conditions do not affect the region of interest of the medium: 5 μm along the radius and 30 μm along the axis. For the numerical solution of the mathematical model, a variant of the HLLC method for the Baer-Nunziato equations was used,

which is described for example in[2], where it was used to simulate high-speed plate collisions. This method has proven successful in solving problems with explicit contact boundaries, in contrast to the well-known HLL method for the Baer-Nunziato equations, which is characterized by a significant circulation viscosity at the contact boundary.

Simulation results

To determine the threshold for crater formation and cavity opening, two-dimensional simulations were carried out using a hydrodynamic code for absorbed energy densities ξ from 10.5 kJ·cm^{-3} to 28 kJ·cm^{-3} with a step of 3.5 kJ·cm^{-3}.

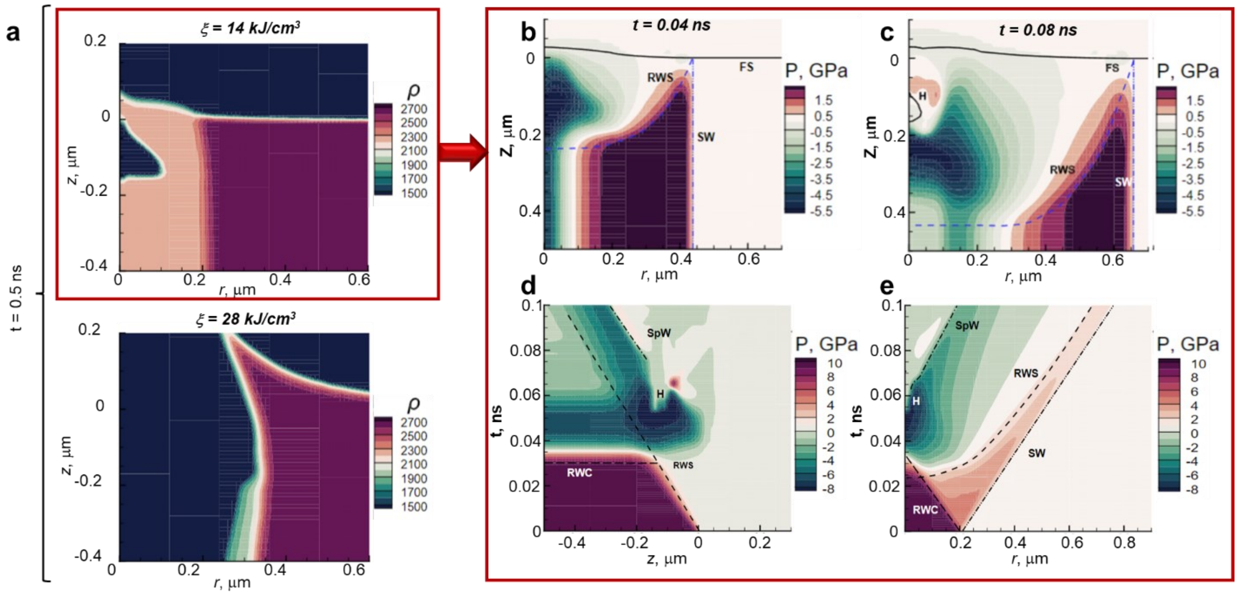

Fig. **S1** Results of 2D HD modeling to investigate the threshold for manifestation of ablation and cavity. **a —** Change in crater structure as a function of irradiation type at absorbed energy density of 14 kJ·cm^{-3} (upper frame) and ξ = 28 kJ·cm^{-3} (lower frame) at a time of 0.5 ns after femtosecond XFEL irradiation; **(b-d)** Two-dimensional pressure maps in the crater region at the formation threshold ξ_{crat} = 14 kJ·cm^{-3} for times **(b) —** 0.04 ns and **(c) —** 0.08 ns (**FS** – free surface, **SW** – radial shock wave, **RWS** – rarefaction wave emanating from the surface, **H** is the resulting cavity). **(d,e)** Overlap of surface **RWS** and radial **RWC** rarefaction waves. Space-time diagrams of the cratering process at ξ_{crat} = 14 kJ·cm^{-3} – **(d)** z-t diagram on the plane $r = 0$ (symmetry axis of the XFEL beam) and **(e) —** *r-t* diagram on the plane $z = -0.15$ (depth of initial cavity formation). **RWS** – rarefaction wave from the surface of the material, **RWC** – radial rarefaction wave, **SW** – radial shock wave, **H** – cavity in the material, **SpW** – spall pulse).

The first signs of the formation of a surface structure leading to the formation of a crater were observed at an irradiance of ξ_{crat} = 14 kJ·cm^{-3}, Fig. S1(a) - top image. Note that the calculated threshold for crater formation in LiF is generally consistent with the results of work[3], where the

first signs of damage in the form of delamination were observed at values of ξ_{damage} = 4 kJ·cm^{-3} per pulse. Figure S1(a) also clearly shows that, in addition to the change in crater shape, a continuous cavity is formed at an irradiance ξ_{cavity} of 28 kJ·cm^{-3}, which is slightly higher than the experimental value found in Figure 3(c): 12-18 kJ·cm^{-3} (in the main article text). This could be due to the fact that the simulations were performed for a layer close to the LiF surface (free boundary ─ LiF vacuum), while the clogging of the cavity is observed at a depth of z ~ 1100 μm.

Let us take a closer look at the process of crater formation. Figures S1(b-d) show the dynamics of crater formation when irradiated with a threshold value ξ_{crat} = 14 kJ·cm^{-3}. Fig. S1(b,c) shows the dynamics of pressure development in the crater area at times of 0.04-0.08 ns. Discontinuity occurs in the thickness of the material, approximately at the height of z = 0.15 μm from the initial free surface (**FS**). Line **FS** denotes the deformed boundary of the material, **RWS** is a hemispherical rarefaction wave propagating from the surface into the substance, the line **H**-denotes the boundary of the gap formed in the material. We argue that the detachment of the material leading to the formation of a crater is caused by the addition of rarefaction waves coming from the surface of the material and from the boundary of the heated zone. The resulting tensile stresses lead to the formation of a crater. To illustrate this statement, consider two space-time diagrams: an r-t diagram on the plane $z = -0.15$ and a z-t diagram on the plane $r = 0$, Fig. S1(d,e). In Figures S1(d) and S1(e), the usual dotted line shows the **RWS** rarefaction wave propagating from the free surface deep into the substance. At the beginning of its motion it is a plane wave, orthogonal to the axis of symmetry, but with time it acquires a curvature. It can be observed that the **RWS** and **SW** waves touch at the **FS** boundary of the sample (see Fig. S1(b,c)), which determines the curvature of the rarefaction wave.

Already at 0.4 ns behind the front of this wave there is an area with considerable negative pressure, about -6 GPa. This fact is related to the arrival of the second rarefaction wave, marked with **RWC** and a long-dotted line, at the same point in space. This rarefaction wave is associated with the decay of the discontinuity at the boundary of the heating jet in the radial direction along the entire heating depth. An element of the same decay is a shock wave **SW** propagating radially from the axis of symmetry, which has no influence on the processes taking place in the region of interest to us. The region of tensile stresses created by the addition of the waves eventually leads to the loss of continuity of the substance, i.e. spallation, denoted by **H**, occurs. After some time, during which the transient processes associated with the loss of continuity take place, spallation

pulses occur, which are labeled with the dashed signature **SpW**. The spallation pulse propagating towards the free surface is not shown due to the complexity of its profile. After the spallation pulse has passed, the pressure in the resulting cavity gradually drops to almost zero.

II. Mathematical model for LiF damage (SPH simulation)

Simulation of deformation and damage of LiF material utilizes the continuum mechanics approach, which is based on the mass, momentum, and energy conservation equations. Those equations are closed by the material models, including the equation of state plus the spallation model and the damage model. A simulated sample may undergo large deformations under high laser energy deposition considered in the main article text. Therefore, it is reasonable to use a mesh-free approach based on the SPH (smoothed particle hydrodynamics) method, since the lagrangian mesh is not able to withstand very large deformations. The representation of material as a set of interacting smoothed particles has some advantages in comparison with the mesh methods. This approach naturally allows to track the movement of existing sample boundaries and formation of the new boundaries via loss of material continuity governed by the spallation model. This greatly simplifies simulation of the material under consideration. A detailed description of the SPH method used in this work can be found in [4,5].

Material models

Equation of state for LiF used in simulations is given in the Mie-Gruneisen form:

$$P = P_r + \gamma\rho(e - e_r), \tag{1}$$

where e is specific internal energy, $P_r(\rho)$ and $e_r(\rho)$ are reference curves, γ is the fixed Gruneisen parameter. We use the linear function $u_s = c + su_p$ for the dependence of the shock wave velocity u_s from the material velocity u_p, where c is the speed of sound in the uncompressed material and s is a fixed coefficient. Taking the notation $x = \rho_0/\rho$, where ρ_0 is the density of the uncompressed material, the reference curves of pressure and internal energy are expressed as follows:

$$P_r(\rho) = \rho_0 c^2 \frac{1 - x}{[1 - s(1 - x)]^2}, \quad e_r(\rho) = \frac{P_r}{\rho_0}\frac{1 - x}{2} \tag{2}$$

The equation of state parameters in Eqs. (1) and (2) used for LiF are presented in Table **S1**.

Table **S1**. Mechanical properties of lithium fluoride LiF[3]

**Mechanical properties**	**LiF**
Density ρ_0	2650 kg/m^3
Shear modulus G	55 GPa
Intact spall strength T_0	1.5 GPa
Intact shear strength σ_i	2.4 GPa
Speed of sound c	5.15 km/s
Shock coefficient s	1.35
Gruneisen parameter γ	0.71

Damage model. Lithium fluoride is a brittle material and is susceptible to damage under the mechanical loading. For LiF we use a damage model of ceramic materials, which has been successfully tested on some ceramics such as boron carbide B_4C, silicon carbide SiC and aluminum nitride AlN[6–8]. In this model, it is assumed that an intact material has the shear strength $\sigma_d = \sigma_i(P)$, below which the material response is elastic. If the equivalent stress σ_{eq} exceeds this limit, the damage is activated as illustrated in Fig. **S**2 and the strength decreases until $\sigma_d = \sigma_f(P) < \sigma_i(P)$, which corresponds to the completely damaged material. Although the physical mechanism of ceramic damage is not fully understood, the kinetics of damage process can be presented by a damage model with empirically selected constants, which can be fitted using the known mechanical properties of material.

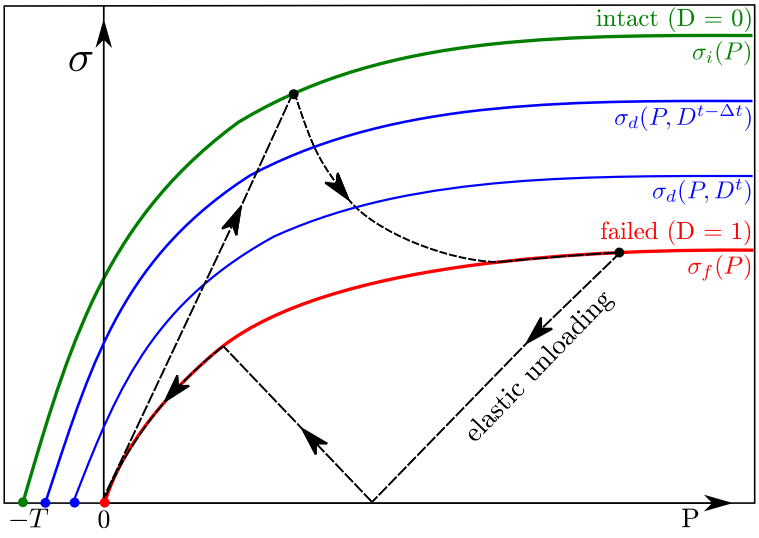

Fig. **S2** Schematic equivalent stress–pressure curves for the damage model of ceramics[6]. The dashed line indicates first elastic compression (linear path) and then inelastic compression producing damage followed by elastic unloading of fully damaged material.

During damage the strength of material $\sigma_d(P)$ is characterized by a model parameter $D \in [0,1]$, which determines the strength of ceramics between the intact state $\sigma_i(P)$ and the completely damaged state $\sigma_f(P)$ as follows:

$$\sigma_d(P) = \sigma_i(P) - D(\sigma_i(P) - \sigma_f(P)) \quad (3)$$

The increment of parameter D at each time step Δt is determined through the increment of plastic deformations $\Delta\varepsilon_p$ during this step. Elastic deformation per step is defined as the difference between the total deformation $\Delta\varepsilon$ and elastic deformation $\Delta\varepsilon_e$:

$$\Delta\varepsilon_p = \Delta\varepsilon - \Delta\varepsilon_e \quad (4)$$

It is assumed that for the transition from an undamaged to a damaged state, the total plastic deformation of the material should be equal to ε_p^f. Evolution of parameter D is defined via the following damage kinetic equation:

$$\frac{dD}{dt} = (1 - D)\frac{1}{\varepsilon_p^f}\frac{d\varepsilon_p}{dt} \quad (5)$$

Then the parameter D can be obtained numerically at the next time step:

$$D^{t+\Delta t} = 1 - (1 - D^t)\exp\left(-\frac{\Delta\varepsilon_p}{\varepsilon_p^f}\right) \quad (6)$$

It should be notice that the damage kinetics with Eq. (5) limits the parameter D by 1. It leads to a smooth deceleration of damage while approaching to its completely damaged state $D = 1$. To estimate the increment of plastic deformations at each time step the next expression is used[9]:

$$\Delta\varepsilon_p = \frac{\hat{\sigma}_{eq} - \sigma_{eq}}{3G}, \quad (7)$$

where G is the shear modulus, $\hat{\sigma}_{eq}$ is the equivalent stress calculated under assumption that all deformations are elastic at the current step, σ_{eq} is the actual equivalent stress (after correction of the deviatoric part of stresses for $\hat{\sigma}_{eq} > \sigma_d$).

Based on Eq. (7) for the increment of plastic deformations, the total plastic deformation required for the transition from an undamaged to a completely damaged state can be written as follows:

$$\varepsilon_p^f = \sum_{D=0}^{1} \Delta\varepsilon_p = \frac{\sigma_i - \sigma_f}{3G} \quad (8)$$

The functional forms for the model $\sigma_i(P)$ and $\sigma_f(P)$ strength curves can be chosen arbitrarily. Their forms are determined by how well they are able to reproduce experimental data. The functional form of the curves $\sigma_i(P)$ and $\sigma_f(P)$ proposed in[10] seems to be quite successful, which is confirmed by our experience in modeling high-strength ceramics such as B_4C, SiC, AlN, etc. Therefore, we use the model strength curves for LiF in the same form:

$$\sigma_\lambda(P) = \begin{cases} \sigma_\lambda^0 + \left(\sigma_\lambda^{max} - \sigma_\lambda^0\right)\{1 - \exp[-\alpha_\lambda(P - P_\lambda)]\}, & P > P_\lambda \\ \sigma_\lambda^0 \dfrac{P}{P_\lambda + T_\lambda}, & P \le P_\lambda \end{cases} \quad (9)$$

$$\alpha_\lambda = \frac{\sigma_\lambda^0}{\left(\sigma_\lambda^{max} - \sigma_\lambda^0\right)(P_\lambda + T_\lambda)} \quad (10)$$

Here the index $\lambda \in \{i, f\}$. The spall strength T is assumed to be linearly dependent on the parameter D as follows: $T = T_0(1 - D)$. The model takes also into account the dependence of material strength on the deformation rate. The detailed description of the ceramic damage model, a step-by-step numerical algorithm, and the results of model verification are presented in[3,6–8].

X-ray pulse absorption model. In our SPH simulations the used X-ray pulse parameters are similar to experimental conditions: the pulse duration is ~20 fs, the photon energy is 9 keV corresponding to the wavelength of $\lambda = 0.138$ nm, the beam FWHM width in the focus is approximately 410 nm, and the average pulse energy is about 81 μJ.

The irradiation by a laser pulse is modeled by setting the specific (per unit mass) energy inside a certain area where the laser pulse energy is deposited:

$$e(x, y, z) = e_0 \exp\left(-\frac{x^2 + y^2}{R^2}\right) \exp\left(-\frac{z}{d_{att}}\right) \quad (11)$$

Here R is the FWHM width of laser beam, d_{att} — is the attenuation length, and the energy factor e_0 is determined by requirement that the integral of $e(x, y, z)$ over the entire volume must be equal to the pulse energy. Since the pulse is very short (~20 fs), the absorption of the X-ray pulse is

assumed to be instantaneous, and heating of the irradiated sample is isochoric. The used attenuation length of about 475 μm is obtained from the database[11] for photon energy of 9 keV.

III. Interatomic potential for LiF in condensed phase (MD simulation)

Ions in ionic crystals like LiF interact with the long-range Coulomb forces, but due to net charge neutrality and periodicity of lattice the effective interatomic potential is attenuated much faster with distance. Using the Ewald summation, it can be represented by the Debye-type potential with some shielding length. To escape time-consuming calculations of the long-range Coulomb forces and perform large-scale molecular dynamics simulations of micrometer-sized samples consisting of many million atoms we have developed a short-range interatomic potential representing the basic properties of condensed phase of LiF, including the normal density of solid at the room temperature, the stress-strain curves of solid LiF, the melting point and densities of solid and liquid at the melting point.

Debye-type pairwise potential for interaction between ions with electrical charges $q_i = \pm 1$ can be written as:

$$V_{ij}(r) = \left[\frac{q_i q_j \mathrm{e}^2}{4\pi\epsilon\, r} \exp\left(-\frac{r}{d}\right) + \frac{a_4}{r^4} + \frac{a_6}{r^6} + \frac{a_8}{r^8}\right] f(r, r_c), \tag{12}$$

where r is interatomic distance, the Coulomb constant $\frac{\mathrm{e}^2}{4\pi\epsilon} = 138.935457645199$ [kJ/mol · nm], d is a fitted Debye length, and a_4, a_6, a_8 are fitting parameters for the additional rapidly decreasing with distance energy terms. The smoothing function

$$f(r, r_c) = \frac{\left[1 - \left(\frac{r}{r_c}\right)^4\right]^4}{1 + \left[1 - \left(\frac{r}{r_c}\right)^4\right]^4}, \tag{13}$$

causes this potential goes smoothly to zero with the interatomic distance approaching the cutoff radius $r_c = 0.875$ nm. The above potential is referred here as Version 1

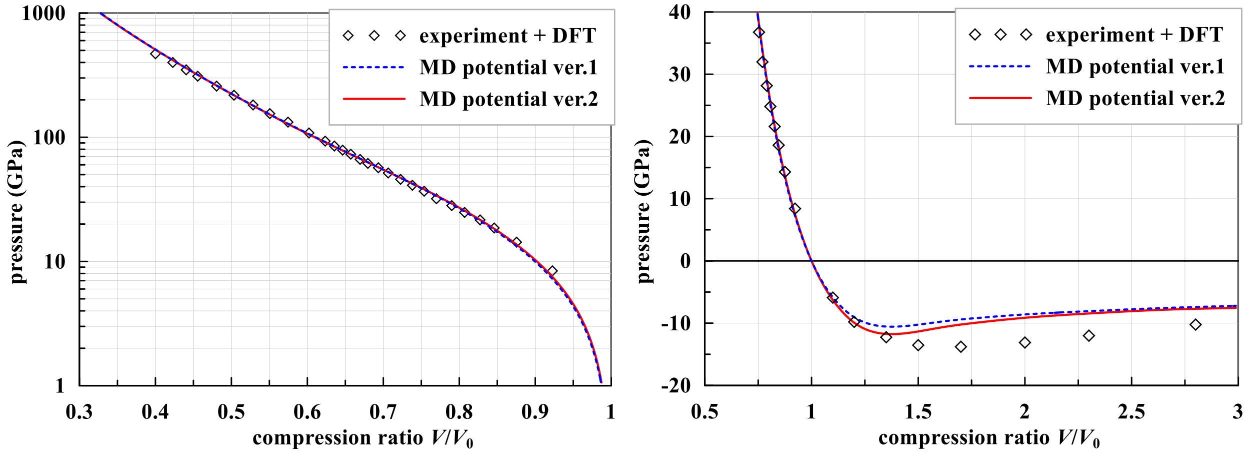

Fig. **S3** Cold pressure curves for compression and stretching of LiF lattice using two versions of interatomic potentials. Experimental data for $V/V_0 < 1$ taken from[12] and DFT data from[13].

The potential parameters were fitted to the known cold pressure curve in a wide range of compression and stretching by using the stress-matching method[14]. Figure **S3** shows the experimental pressures[12] at compression and DFT calculated data[13] at stretching of solid LiF together with pressures provided by the fitted potential with parameters listed in Table **S2**. The Debye length $1/d = 4.9616735392993485$ nm^{-1} was found together with other parameters by the fitting.

Table **S2** Fitting parameters for both versions of Debye-type potentials

Parameter	F-F	Li-Li	Li-F
a_4 [kJ/mol · nm^4]	-2.8948849974589106E-2	-3.3835629437645717E-2	1.0977735841792622E-2
a_6 [kJ/mol · nm^6]	-2.0884485104425644E-4	7.5501774001012140E-4	1.9166106664994221E-3
a_8 [kJ/mol · nm^8]	2.6253652344807973E-6	2.6798551354309170E-10	3.4901705879549604E-12

To avoid calculation of exponential function it is approximated by a rational function as follows:

$$V_{ij}(r) = \left[\frac{q_i q_j \mathrm{e}^2}{4\pi\epsilon\, r} \frac{1}{1 + \frac{d}{r}\left(b_1 + b_2 \left(\frac{d}{r}\right)^2\right)} + \frac{a_4}{r^4} + \frac{a_6}{r^6} + \frac{a_8}{r^8} \right] f(r, r_c), \quad (14)$$

where $b_1 = 1.15$ and $b_2 = 0.569$ are fitting parameters of the rational function representing the Debye-type screening. This potential is referred here as Version 2. The fitting parameters a_4, a_6, a_8 and the Debye length d are the same as in Version 1.

Figure **S3** demonstrates that the stress-strain curves from both presented potentials are almost identical. It is not surprising since the virials of interatomic forces for all pairs of atoms shown in Fig. **S4** are also very close to each other.

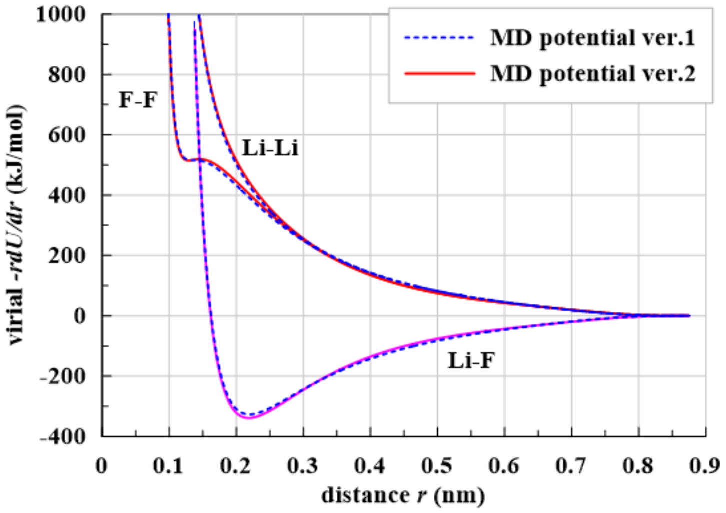

Fig. **S4** Virials of pairwise forces between F-F, Li-Li, and Li-F atoms for two versions of potentials.

Both presented above potentials reproduces the experimental difference of 25% between the densities of molten and solid LiF at the melting point. Such peculiar feature of LiF is important for cavitation of stretched metastable liquid during fast cooling in isochoric conditions (inside the channel having the fixed solid wall) as demonstrated in the main article text. It was surprisingly found that the potential Version 1 provides the melting temperature of 910 K, which is notably lower than $T_m = 990$ K obtained for the potential Version 2. Since it is closer to the experimental $T_m = 1118$ K for LiF, the potential Version 2 was used in all our productive simulations presented in the main article text.

IV. Supplementary References

1. Baer, M. R. & Nunziato, J. W. A two-phase mixture theory for the deflagration-to-detonation transition (DDT) in reactive granular materials. *Int. J. Multiph. Flow* **12**, 861–889 (1986).

2. Chuprov, P., Utkin, P. & Fortova, S. Numerical simulation of a high-speed impact of metal plates using a three-fluid model. *Metals (Basel).* **11**, 1233 (2021).

3. Makarov, S. *et al.* Damage threshold of LiF crystal irradiated by femtosecond hard XFEL pulse sequence. *Opt. Express* **31**, 26383–26397 (2023).

4. Egorova, M. S., Dyachkov, S. A., Parshikov, A. N. & Zhakhovsky, V. V. Parallel SPH modeling using dynamic domain decomposition and load balancing displacement of Voronoi subdomains. *Comput. Phys. Commun.* **234**, 112–125 (2019).

5. Parshikov, A. N. & Medin, S. A. Smoothed Particle Hydrodynamics Using Interparticle Contact Algorithms. *J. Comput. Phys.* **180**, 358–382 (2002).

6. Dyachkov, S. A. *et al.* Explicit failure model for boron carbide ceramics under shock loading. *J. Appl. Phys.* **124**, (2018).

7. Andriyash, A. V. Arinin, V. A. Dyachkov, S. A. et al. Long-term evolution of spherical shell with boron carbide layer after explosive compression. *J. Appl. Phys.* **126**, 085903 (2019).

8. Grigoryev, S. Yu., Dyachkov S.A., P. A. N. & V., Z. V. Failure model with phase transition for ceramics under shock loading. *J. Appl. Phys.* **13**, 125106 (2022).

9. Wilkins, M. L. *Computer Simulation of Dynamic Phenomena*. (Springer Berlin, Heidelberg, 1999).

10. Johnson, G. R. *et al.* Response of aluminum nitride (including a phase change) to large strains, high strain rates, and high pressures. **94**, (2003).

11. Henke, B. L. L., Gullikson, E. M. M. & Davis, J. C. C. X-Ray Interactions: Photoabsorption, Scattering, Transmission, and Reflection at E = 50-30,000 eV, Z = 1-92. *At. Data Nucl. Data Tables* **54**, 181–342 (1993).

12. Dong, H. *et al.* Compression of lithium fluoride to 92 GPa. *High Press. Res.* **34**, 39–48 (2014).

13. Smirnov, N. A. Ab initio calculations of the thermodynamic properties of LiF crystal. *Phys. Rev. B* **83**, 014109 (2011).

14. Zhakhovskii, V. V., Inogamov, N. A., Petrov, Y. V., Ashitkov, S. I. & Nishihara, K. Molecular dynamics simulation of femtosecond ablation and spallation with different interatomic potentials. *Appl. Surf. Sci.* **255**, 9592–9596 (2009).
