# Supplementary material for: Formation of high-aspect-ratio nanocavity in LiF crystal using a femtosecond X-ray free-electron laser pulse
Source: Nat Commun. 2025 Dec 17;16:11504. doi: 10.1038/s41467-025-66481-6 (PMC12749636; doi:10.1038/s41467-025-66481-6)
Supplement: Supplementary file 2 — Description of Additional Supplementary File [file 41467_2025_66481_MOESM2_ESM.pdf]

The Description of Additional Supplementary Files

Supplementary Video 1: - The full-scale MD simulation of the 2D distributions of the damage and the equivalent von Mises stress (from Fig. 5a).

Supplementary Video 2: - MD simulation with a detailed evolution of the processes of crystallization and cavitation in the cylindrical channel with the fixed solid walls (from Fig. 5b).
